# Context-Dependent Enhancer Selection Confers Alternate Modes of Notch Regulation on *argos*

**DOI:** 10.1128/MCB.01045-13

**Published:** 2014-02

**Authors:** Benjamin E. Housden, Ana Terriente-Felix, Sarah J. Bray

**Affiliations:** Department of Physiology, Development and Neuroscience, University of Cambridge, Cambridge, United Kingdom

## Abstract

Wiring between signaling pathways differs according to context, as exemplified by interactions between Notch and epidermal growth factor receptor (EGFR) pathways, which are cooperative in some contexts but antagonistic in others. To investigate mechanisms that underlie different modes of cross talk, we have focused on *argos*, an EGFR pathway regulator in Drosophila melanogaster which is upregulated by Notch in adult muscle progenitors but is repressed in the wing. Results show that the alternate modes of cross talk depend on the engagement of enhancers with opposite regulatory logic, which are selected by context-determining factors. This is likely to be a general mechanism for enabling the wiring between these pathways to switch according to context.

## INTRODUCTION

Notch signaling produces a large array of cellular outputs despite its relatively simple transduction pathway (1–5). These diverse outcomes are reflected in the target genes regulated in different cell types and in the impact these targets have on cellular functions (6–10). Indeed, there are many examples of direct Notch target genes that are regulated in distinct spatial and temporal patterns following pathway activation (11, 12). While it is well established that the response to Notch signaling is highly context dependent, our knowledge of the molecular mechanisms leading to this is limited. Understanding how such differential regulation is achieved will be important for deciphering regulatory networks involved in controlling developmental decisions. For example, in adult muscle progenitors (AMPs), the epidermal growth factor receptor (EGFR) and Notch pathways appear to function cooperatively, and several EGFR pathway genes are directly upregulated in response to Notch activation (6). In contrast, wing vein development involves an antagonistic relationship between EGFR and Notch, with EGFR promoting vein development and Notch preventing it (13). In this context, expression of the EGFR pathway gene, *rhomboid*, is inhibited by Notch activity (14, 15). One fundamental question is how the distinct interactions between the two pathways are conducted.

The key DNA binding protein in the Notch pathway is CSL. Binding of ligand (Delta or Serrate in Drosophila melanogaster) to the Notch receptor leads to the release of the Notch intracellular domain (Nicd) into the cytoplasm. This fragment is then able to interact directly with a transcription factor of the CSL family [Suppressor of Hairless; Su(H) in Drosophila]), leading to activation of target gene expression (16, 17). As with other genes directly regulated by Notch, CSL is recruited to binding motifs in a known enhancer region of the *argos* gene, one of the EGFR pathway genes which is upregulated in the AMPs (6). What is less clear is whether these sites are no longer responsive in contexts, such as the wing, which exhibit antagonistic cross talk with EGFR and how this alternate mode of regulation is implemented.

Here, we have focused on *argos* to investigate the mechanisms responsible for switching the mode of regulatory cross talk between signaling pathways. We show that this relies on separable enhancers within the intron of *argos*; an AMP enhancer which is positively regulated by Notch and two wing pouch enhancers that direct expression in the wing veins. Of those, one receives input from Notch through the E(spl) basic helix-loop-helix (bHLH) repressors, explaining the inhibitory effects. The second wing enhancer is regulated by the EGFR pathway, via the inactivation of the repressor Capicua (18), and receives no input from the Notch pathway apart from through its impact on EGFR pathway activity. Interestingly, each enhancer becomes refractive to their normal inputs outside their respective contexts, and different modes of regulation cannot be explained simply by additive effects through the three enhancers. Instead, our data suggest that context-determining factors regulate accessibility of different enhancers in each tissue. These results demonstrate how opposite outputs can be generated by a signaling pathway through the context-specific engagement of enhancers with different regulatory logic.

## MATERIALS AND METHODS

### Fly lines.

All fly lines are described in FlyBase, and where possible, established reagents were used for which functionality was already established in previous publications: *1151-Gal4* (19), *SalpE80-Gal4* (20), *argos*(*p*)-*lacZ* (21), *argos1-lacZ* (6), upstream activation sequence (UAS)-*Su*(*H*)-*VP16* (11), UAS-*Nicd* (22), UAS-*Notch*-RNAi (Bloomington no. 7078) (23), UAS-*HLHm*β (24), UAS-*HLHmβ-VP16* (25), UAS-*Twist* (26), UAS-*sd*-RNAi (TRiP-29352) (27), UAS-*salm*-RNAi (VDRC-101052) (28), UAS-*Dp*-RNAi (VDRC-12722) (www.genomeRNAi.de), and UAS-*vvl*-RNAi (TRiP-26228) (29). For untested RNA interference (RNAi) reagents, functionality was established by examining their respective wing phenotypes and comparing them to expected effects based on previous literature (see Fig. 5A, C, E, G, I, K, and M): UAS-*cic*-RNAi (VDRC-103805), UAS-*grh*-RNAi (TRiP-28820), UAS-*Gug*-RNAi (VDRC-107413), and UAS-*dwg*-RNAi (VDRC-100245).

### Construction of reporter lines.

*argos1-lacZ* was reported previously and consists of a 3.1-kb genomic fragment (3L:16465839..16468927). *argos2-GFP* (3L:16468929..16472278) and *argos3-GFP* (3L:16473095..16474078) reporters were produced by amplifying fragments from genomic DNA by PCR using the following primers: argos2-fwd (GTTAGACGAGACGGATGGATG), argos2-rvs (TTATTCAATGCGATTCGAAGG), argos3-fwd (GAGATGAAAGTTTATAG), and argos3-rvs (ACCAATGAAACCAACAACTGG). Note that *argos3* is the same fragment as that reported in reference 18. Fragments were cloned into the pGreenRabbit vector (23) and inserted into the attP 86Fb injection line (30). *argos2cic-GFP* and *argos3cic-GFP* were produced using site-directed mutagenesis to alter Cic motifs in *argos2-GFP* and *argos3-GFP* before injecting them into the same *attP* stock as the wild-type reporters. Conserved Cic motifs, TGAATG(G/A)A, were altered to TGCGTGTG, a mutation previously shown to remove Cic regulation (18, 31).

### Immunostaining.

Immunostaining was performed, as described previously (32), using the following primary antibodies: mouse anti-β-galactosidase (Developmental Biology Hybridoma Bank), rabbit anti-green fluorescent protein (anti-GFP) (Molecular Probes), and goat anti-GFP (Abcam). Fluorescent images were obtained using either a Zeiss Axiophot microscope or a Nikon Eclipse C1 confocal microscope. Images were analyzed using ImageJ and Adobe Photoshop.

### X-Gal staining.

Larval heads were dissected and fixed in 2.5% glutaraldehyde for 7 min before incubation in 5-bromo-4-chloro-3-indolyl-β-d-galactopyranoside (X-Gal) staining solution containing 1 ml 10× phosphate-buffered saline (PBS), 100 μl 1 M MgCl_2_, 300 μl Triton X-100, 320 μl 0.1 M K_4_[Fe^2+^(CN)_6_], 320 μl 0.1 M K_3_[Fe^3+^(CN)_6_], 8 ml double-distilled H_2_O, and 100 μl 20% X-Gal in dimethylformamide (DMF) at 37°C until staining developed. Wing discs were then removed and mounted in 70% glycerol. Discs were imaged using a Zeiss Axiophot microscope and analyzed using ImageJ and Adobe Photoshop.

## RESULTS

### Notch regulates *argos* through context-specific enhancers.

**FIG 1:**
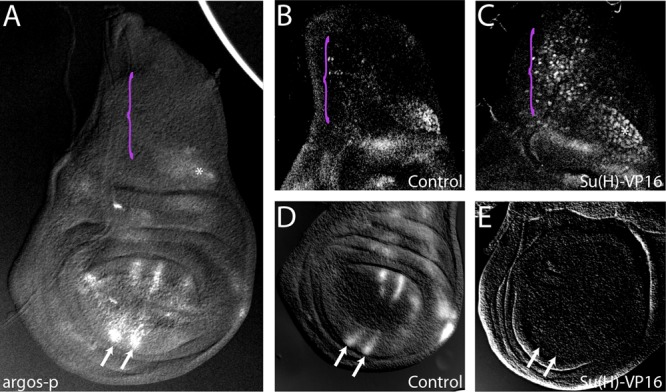
Tissue-specific regulation of *argos* by Notch. (A to E) Expression of the *argos*(*p*)-*lacZ* reporter in the wild type (A, B, and D) or with ectopic Notch pathway activity (Su(H)-VP16) in the AMPs (C) or the wing pouch (E). Purple brackets indicate the region containing AMPs, and arrows indicate the wing veins.

To understand the relationship between Notch activation and pathway output, we focused on the regulation of one Notch target gene, *argos*. Using flies carrying an insertion of *lacZ* close to the promoter of *argos* [*argos*(*p*)-*lacZ*], which recapitulates the *argos* expression pattern (21) (Fig. 1A), we investigated the differential response to Notch signaling in AMPs and wing pouch using the GAL4 system to direct expression of a constitutively active version of Su(H), Su(H)-VP16, in different regions. When expressed in AMPs (using *1151-Gal4*), Su(H)-VP16 caused an increase in *argos*(*p*)-*lacZ* expression (Fig. 1B and C). In contrast, when Su(H)-VP16 was expressed in the wing pouch (using *SalpE80-Gal4*), *argos*(*p*)-*lacZ* expression was strongly repressed (Fig. 1D and E).

**FIG 2:**
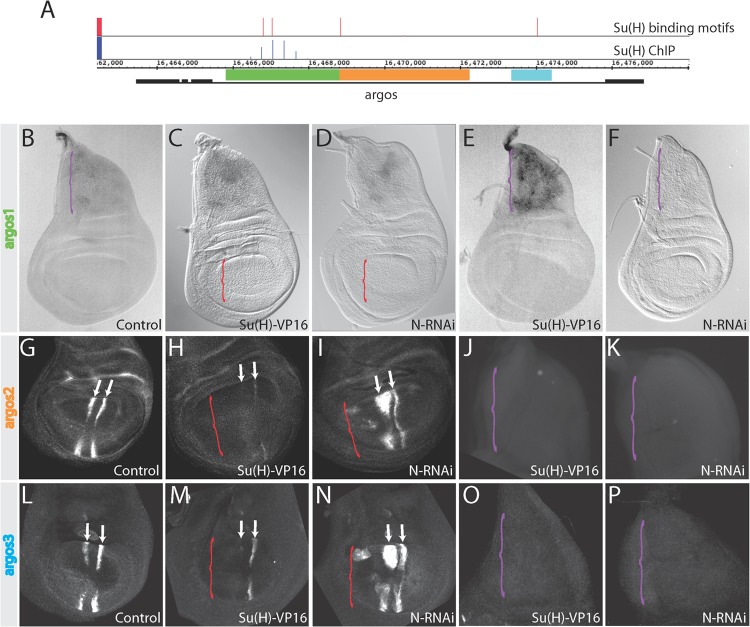
Distinct Notch regulation is mediated by separable enhancers. (A) Schematic of the *argos* gene. Black boxes indicate exons and black lines indicate introns. Red bars, conserved Su(H) binding motifs where the height of the bar indicates the patser score (5.1 to 9.8). Blue bars, Su(H)-bound regions identified by ChIP in DmD8 cells (0.3- to 1.4-fold enrichment on a log_2_ scale). *argos1*, *argos2*, and *argos3* enhancer regions are indicated by the green, orange, and blue rectangles, respectively. (B to P) Expression from the indicated enhancers in wing discs under different conditions: *argos1* (B to F), *argos2* (G to K), or *argos3* (L to P) reporters in wild-type (B, G, and L), Notch pathway activation [Su(H)-VP16] (C, E, H, J, M, and O), or Notch suppression (N-RNAi) (D, F, I, K, N, and P) conditions. Brackets indicate regions where Notch pathway activity was manipulated: panels C, D, H, I, M, and N, wing pouch expression using *salpE80-Gal4* (red brackets); panels E, F, J, K, O, and P, AMP expression using *1151-Gal4* (purple bracket). In panels B to F, whole discs with both thorax (AMPs) and wing pouch are shown; in panels G to I and L to N, wing pouch only is shown; in panels J to K and O to P, thorax (AMPs) only is shown. Arrows mark the L3 and L4 veins.

Analysis of Su(H)-occupied regions in chromatin from Drosophila cells identified a Su(H) binding site in the intron of the *argos* gene (Fig. 2A) (6). A fragment spanning this region (*argos1*) confers Notch responsiveness when cloned upstream of a *lacZ* reporter gene (Fig. 2B, E, and F) (6). However, *argos1-lacZ* expression is restricted to AMPs, and it appears incapable of responding to changes in Notch activity in the wing pouch (Fig. 2C and D). No expression from this enhancer was detected even under conditions where ectopic Su(H)-VP16 was expressed (Fig. 2C), despite the fact that its response in AMPs depends on direct binding of Su(H) (6).

To investigate what mediates the response to Notch in the wing pouch, two other regions were tested for enhancer activity based on conservation in the *argos* gene. Both generated wing pouch expression. *argos2*, a 3.3-kb fragment adjacent to *argos1*, was sufficient to drive expression in a pattern similar to that of *argos*(*p*)-*lacZ* in the wing pouch but not in AMPs (Fig. 2A, orange bar). A third region within the *argos* intron (*argos3*) was similarly able to drive expression in the wing pouch (Fig. 2A, blue bar). Note that *argos3* is the same wing enhancer fragment as that reported recently (18). Therefore, we tested the response of both enhancers to changes in Notch signaling, first using Su(H)-VP16. In the wing pouch, expression of *argos2-GFP* and *argos3-GFP* were both reduced by Su(H)-VP16. Conversely, both gave elevated expression when Notch activity was reduced by RNAi (Fig. 2H to I, M, and N). However, this upregulation was restricted to the region where the enhancer is normally active, the L3 and L4 vein region stripes. There was no spread in expression through the rest of the domain where Notch was reduced. This indicates, first, that other factors help to limit the activity of the enhancer to the provein regions and, second, that Notch signaling can influence *argos2-GFP* and *argos3-GFP* expression even within the wing veins where Notch is thought to be inactive under wild-type conditions. Possible explanations for the upregulation throughout the vein region in Notch RNAi-treated discs are either that there is normally a low level of Notch activity within the vein, which dampens the *argos2-GFP* and *argos3-GFP* expression there, or that the loss of Notch activity in intervein regions influences EGFR activity nonautonomously (e.g., via derepression of *rhomboid*, which would enhance production of EGFR ligands) (14). Finally, neither *argos2-GFP* nor *argos3-GFP* showed any increase in expression when Notch activity was perturbed in AMPs (Fig. 2J, K, O, and P). These data demonstrate that the opposing effects of Notch on *argos* expression in the wing pouch and AMPs are mediated through separable enhancer elements (*argos1* in AMPs, *argos2* and *argos3* in the wing pouch), and that these enhancers are unresponsive to Notch outside their normal context of operation.

### HLHmβ mediates wing pouch repression of *argos* downstream of Notch.

**FIG 3:**
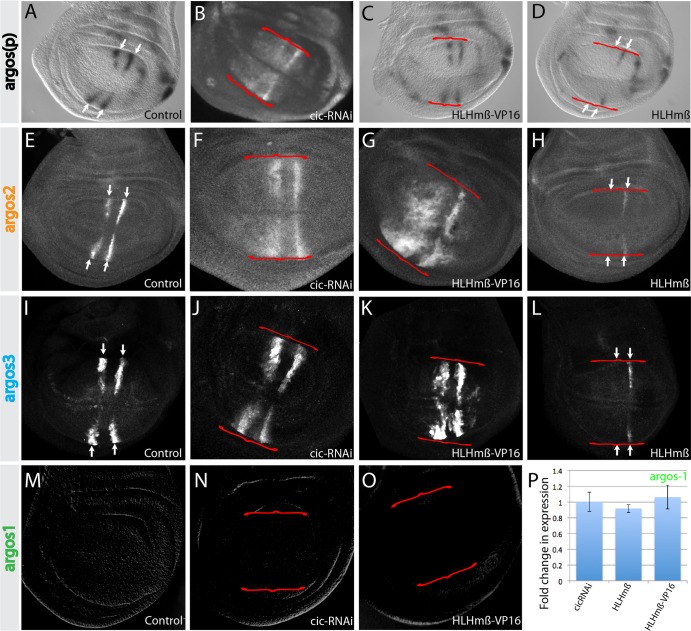
Wing pouch enhancers mediate inputs from Notch and EGFR pathways. (A to O) Expression from *argos*(*p*)-*lacZ* (A to D), *argos2-GFP* (E to H), *argos3-GFP* (I to L), or *argos1-lacZ* (M to O) reporters in wild-type discs (A, E, I, and M) or in the presence of *cic*-RNAi (B, F, J, and N), HLHmβ-VP16 (C, G, K, and O), or HLHmβ (D, H, and L). (P) Quantification of *argos1-lacZ* reporter expression in the AMPs under the conditions indicated. Data are averages from at least 5 discs per genotype, and error bars represent standard errors of the means. Red brackets indicate the region of transgene expression, and arrows mark the L3 and L4 veins.

Given that Su(H) is thought to act as a transcriptional activator in the presence of Notch signaling, it is likely that the observed repression in the wing pouch is due to an indirect mechanism. Previous studies have suggested that the direct Notch target HLHmβ represses another EGFR pathway gene, *rhomboid*, in the wing pouch (14). HLHmβ is expressed in a pattern complementary to that of *argos* in the wing pouch, making it a plausible candidate to mediate *argos* repression downstream of Notch. We tested this possibility by overexpressing HLHmβ. As predicted, this led to a decrease in *argos*(*p*)-*lacZ* expression in the wing pouch (Fig. 3A and D). Similarly, both *argos2-GFP* and *argos3-GFP* reporters were also repressed when HLHmβ was overexpressed (Fig. 3E, H, I, and L), suggesting that HLHmβ acts through these enhancers. To complement these experiments, we examined the consequences of expressing HLHmβ-VP16, in which the terminal WRPW repressor domain is replaced by the VP16 transcriptional activator sequence. Both *argos2-GFP* and *argos3-GFP* were upregulated, consistent with them mediating effects from HLHmβ (Fig. 3G and K). However, no change in *argos*(*p*)-*lacZ* expression was detected in the presence of HLHmβ-VP16 (Fig. 3C), suggesting that additional mechanisms limit the actions of HLHmβ-VP16 at the endogenous gene. Furthermore, no effect of HLHmβ-VP16 on *argos1-lacZ* was detectable in the wing pouch (Fig. 3M and O), and there was no change in either *argos1-lacZ*, *argos2-GFP*, or *argos*(*p*)-*lacZ* expression following expression of HLHmβ or HLHmβ-VP16 in the AMPs (Fig. 3P and data not shown). These results support a model in which HLHmβ acts downstream of Notch in the wing pouch to repress *argos* via the *argos2* and *argos3* enhancers but has no effect in AMPs.

### *argos* integrates inputs from EGFR as well as the Notch pathway.

The EGFR pathway is important for patterning the wing veins during larval and pupal stages (13) and has previously been shown to regulate *argos* expression by attenuating activity of the widely expressed transcriptional repressor Capicua (Cic) (18, 35). Ablating Cic, using RNAi, results in increased *argos*(*p*)-*lacZ* expression in vein primordia and some ectopic expression in intervein regions (Fig. 3B) (18, 35). Thus, it is possible that all effects of Notch/HLHmβ on *argos* expression in the wing pouch are indirect via changes in EGFR pathway activity that in turn impinge on Cic.

To investigate this possibility, the consequences on individual *argos* enhancers of depleting Cic were analyzed under similar conditions. *argos2-GFP* expression was increased in vein primordia, and ectopic expression was observed in intervein regions, resembling the response of *argos*(*p*)-*lacZ* (Fig. 3F). *argos3-GFP* exhibited similar, albeit weaker, ectopic activity (Fig. 3J). However, neither fully recapitulated the effects of depleting Notch activity, suggesting that Cic-independent regulation is also involved. Furthermore, *argos1* was unresponsive to *cic*-RNAi in the wing pouch (Fig. 3N); likewise, *argos1-lacZ*, *argos2-GFP*, and *argos*(*p*)-*lacZ* were not affected when *cic*-RNAi was expressed in AMPs (Fig. 3P and data not shown). Thus, *argos2* and *argos3*, but not *argos1*, receive input from the EGFR pathway at least in part via inactivation of Cic in the wing pouch, but this regulation is limited to certain contexts.

**FIG 4:**
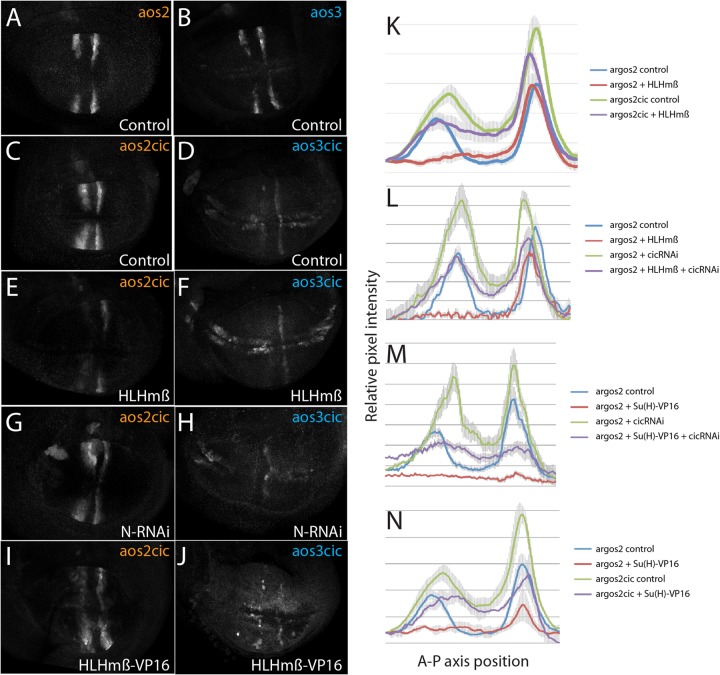
Relationship between Notch and Capicua regulation. (A to D) Expression from *argos2-GFP* (A), *argos3-GFP* (B), *argos2cic-GFP* (C), and *argos3cic-GFP* (D) under wild-type conditions is shown. (E to J) Expression from *argos2cic-GFP* (E, G, and I) and *argos3cic-GFP* (F, H, and J) in the presence of the indicated transgenes driven by *SalpE80-Gal4*. Note that due to low levels of expression from the *argos3cic-GFP* reporter, higher exposure settings were required for imaging, and background fluorescence appears higher in these images. (K to N) Quantification of *argos2-GFP* expression across the center of the wing pouch (anterior left to posterior right) in the presence (red and purple lines) or absence (blue and green lines) of HLHmβ or Su(H)-VP16 overexpression, either with wild-type Cic input (red and blue lines) or with inhibited Cic input using *cic*-RNAi or mutated Cic binding motifs (green and purple lines). Lines represent average expression quantified from a minimum of five discs per genotype; error bars indicate standard errors of the means.

**FIG 5:**
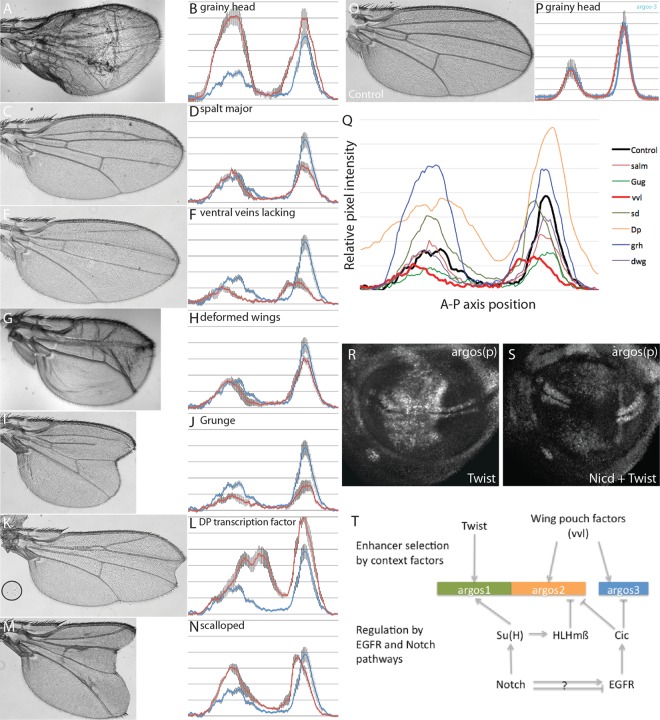
Regulators of *argos* enhancer activity in the wing pouch. (A to O) Analysis of *argos2-GFP* expression (B, D, F, H, J, L, and N) or adult wing phenotype (A, C, E, G, I, K, and M) in the presence of RNAi targeting the indicated genes driven using *SalpE80-Gal4* or under wild-type conditions (O). (P) Quantification of *argos3-GFP* under wild-type conditions (blue line) or in the presence of *grh*-RNAi (red line). Quantification was performed as described for Fig. 4K to N. (Q) Summary for quantification of *argos2-GFP* expression in the presence of RNAi for the indicated genes. (R and S) *argos*(*p*)-*lacZ* expression in the wing pouch in the presence of Twist overexpression (R) or Twist and Nicd overexpression (S). (T) Model of *argos* regulation through the three identified enhancers.

To further test Cic's contribution to the repression from Notch, the Cic binding motifs within *argos2* and *argos3* were mutated. For *argos2*, consequences were similar to Cic knockdown with ectopic expression detected in interveins (Fig. 4A and C), suggesting that Cic is involved in restricting expression to vein regions. However, the levels of expression around the vein regions were not elevated to the extent seen with Notch RNAi, suggesting that additional Notch inputs exist. Unexpectedly, when the 3 conserved Cic sites were removed from within *argos3*, the expression levels were considerably decreased within the veins as well as being derepressed in the regions flanking the dorsal ventral boundary (Fig. 4B and D). This differed from the consequences of eliminating 2 additional Cic-related motifs in *argos3*, which resulted in an enhancer with more widespread derepression (18), and suggests that the remaining motifs in *argos3-Cic* are sufficient to confer some residual Cic-mediated repression. The pattern also differed from that of wild-type *argos3*, which retained high levels of expression when *cic* expression was reduced by RNAi. One possibility is that the mutations in *argos3-Cic* also eliminated activating inputs. Grh is a transcriptional activator whose binding motifs sometimes overlap those of Cic (34). Indeed, examination of *argos3* did reveal the presence of putative Grh binding motifs overlapping the mutated Cic sites. However, although *grh*-RNAi produced phenotypic consequences on wing vein development (Fig. 5A), it had no effect on *argos3-GFP* wing pouch expression (Fig. 5P), making it unlikely that loss of Grh regulation accounts for the reduced expression from the mutated *argos3*.

### Integration of the Notch and EGFR signals.

Although *argos* relies on inputs from both Notch and EGFR pathways to achieve its expression pattern in the wing pouch, these inputs could be integrated via several mechanisms. For example, as Notch alters activity of the EGFR pathway by regulating *rhomboid*, all of its effects on *argos* could be mediated by Cic. An alternative possibility is that there are independent inputs from the two pathways, decoded at the enhancer level. The fact that mutations in Cic binding motifs did not fully recapitulate the consequences from inhibiting Notch suggest that the latter is plausible. To distinguish between the possibilities, we compared the effects of perturbing the Notch input [overexpression of HLHmβ, HLHmβ-VP16, Su(H)-VP16, or N-RNAi] in the presence or absence of the EGFR input (*cic*-RNAi or mutation of Cic binding sites).

If Notch acts indirectly via the EGFR pathway, we would expect stimulation of the Notch input to have no effect in the absence of Cic. We found, however, that the combination of *cic*-RNAi and either Su(H)-VP16 or HLHmβ gave a phenotype intermediate between either treatment alone on *argos2-GFP*, consistent with independent inputs from the two pathways. Similar results were obtained using several different treatments to alter Notch and EGFR inputs (Fig. 4C, E, G, I, and K to N and data not shown), confirming that Notch can regulate *argos*(*p*)-*lacZ* or *argos2-GFP* independently of the EGFR pathway.

Interestingly, similar experiments with *argos3* gave different results. As shown above, removal of Cic binding sites from *argos3* caused a strong reduction in the expression, although some pattern could still be detected (*argos3-Cic*) (Fig. 4D). This mutated enhancer also appeared to have lost the repression by the Notch pathway. No increase in expression was detected with N-RNAi (Fig. 4H), and there was no further inhibition by HLHmβ (Fig. 4F) or activation by HLHmβ-VP16 in the wing veins (Fig. 4J), although in advanced discs, where there were ectopic sensory organs developing, the enhancer became more strongly upregulated in presumptive sensory organ precursors (data not shown). We attribute the latter to the fact that this enhancer can respond to proneural proteins, while it has in general lost the ability to respond to changes in Notch activity. On this basis, we conclude that the effects of Notch on *argos3* are largely mediated indirectly through the EGFR pathway acting via the Cic binding sites.

### Context-specific factors determine the effect of Notch signaling.

Our data suggest that separate enhancers account for the opposing regulation of *argos* by Notch, with *argos1* mediating positive input by Su(H) and *argos2* mediating negative input via bHLH repressors. Furthermore, each enhancer must be available only to the requisite regulators in specific contexts. For example, we demonstrated previously that the bHLH transcription factor Twist acts as a context-determining factor in AMPs, where it is required for activation of Notch target genes, including *argos*, conferring responsiveness on the *argos1* enhancer (36). We hypothesize that similar factors will enable HLHmβ and Cic to bind and regulate *argos2* and *argos3* in the wing pouch while leaving *argos1* inaccessible. It is likely that such a factor would be widely expressed (to generate the broad upregulation throughout the wing pouch in the presence of *cic*-RNAi). Therefore, we ablated expression of 7 transcription factors, which were reported to have widespread wing pouch expression, and analyzed the consequences on *argos2-GFP* (Fig. 5A to O and Q). Of those tested, only 2, *vvl* and *Gug*, led to a clear reduction in *argos2-GFP* consistent with the possibility that they are positive regulators. As Grunge/atrophin (Gug) is thought to function as a corepressor, it is likely to mediate its effects indirectly, making the POU factor *ventral-veinless* (*vvl*) the most likely candidate. Other gene knockdowns modified expression in a more localized manner, probably due to their role in vein specification (e.g., *salm*) (Fig. 5C and D), and one resulted in strong derepression (*Dp*) (Fig. 5K to L). We note that many gave stronger phenotypes in adult wings than in the imaginal discs, indicating likely roles during later stages of wing development.

To determine what would happen if two different context-specific factors were combined, the consequences on *argos*(*p*)-*lacZ* of ectopically expressing Twist in the wing pouch were examined. Under normal conditions, Twist expression led to upregulation of *argos*(*p*)-*lacZ*, consistent with it activating expression from the *argos1* enhancer under these conditions of low Notch activity (Fig. 5R). However, when Nicd was coexpressed with Twist, it produced a mixed response. *argos*(*p*)-*lacZ* expression was largely repressed, but some regions of ectopic expression remained (Fig. 5S). This suggests that *argos*(*p*)-*lacZ* is able to respond to both the positive inputs from Notch through *argos1* and the repressive inputs from increased HLHmβ through *argos2* under these conditions.

## DISCUSSION

Our analysis of tissue-dependent responses to Notch demonstrates that, in *argos*, these are determined at the level of specific enhancers. These respond either to Su(H) or to the bHLH repressors downstream of Notch, giving rise to different consequences on *argos* expression and explaining how the logic of signaling pathway cross talk can be switched. Indeed, the different modes of *argos* regulation correlate with the relationship between Notch and EGFR pathways, with cooperative cross talk occurring in the AMPs, where the enhancer directly regulated by Su(H) is active, and antagonistic cross talk taking place in the wing pouch, where the repressive enhancer regulated by bHLH operates. Similar distinctive enhancers may also operate at different stages in development, where Notch first activates and then represses the expression of a gene via independent regulatory elements (37, 38). In both cases it is likely that context-determining factors will alter the ability of specific enhancers to respond to distinct Notch inputs. These will then dictate how signaling pathways will act on the cognate gene, depending on which regulatory elements they make available.

Several observations, such as the inability of HLHmβ or HLHmβ-VP16 to alter expression of *argos*(*p*)-*lacZ* when expressed in the AMPs, suggest that, like Su(H), HLHmβ can occupy its binding sites only when the enhancer becomes accessible. Consistent with this possibility, another HLH family transcriptional repressor, Hairy, was shown to bind and repress only those enhancers that had been rendered accessible by prior binding of other factors (39). Alternatively, HLHmβ may still be capable of binding to its site in *argos2* but lacks the ability to mediate long-range repression, restricting its effects to transcription factors bound within the same vicinity, as observed for short-range repressors regulating *even-skipped* enhancers (40). Given that Hairy bHLH repressors can mediate long-range as well as short-range repression, this explanation seems unlikely (39, 41). Furthermore, as studies of other bHLH factors, such as Myc, argue that they can only bind to chromatin in open conformations (42), the model in which enhancer accessibility is regulated seems the more probable explanation.

Thus, the context-dependent response of *argos* to Notch could be explained by a two-stage model (Fig. 5T). Key determining factors, such as Twist in the AMPs (36) or Vvl in the wing pouch, would first regulate the accessibility of different enhancers in the *argos* intron. This would enable the second stage, which integrates the effects of Notch and EGFR. For example, in the wing pouch, multiple binding sites for the repressor Cic keep the gene repressed, except in regions where EGFR is active. Superimposed on this is the additional regulation from the E(spl)bHLH repressors, acting downstream of Notch to fine-tune the expression patterning within this active domain. Such a model is broadly consistent with two general principles proposed previously for gene regulation by signaling pathways (43). The first is the reliance on cooperation with context-determining transcription factors, fulfilled here by the requirements for Twist or Vvl. The second is the pivotal role played by repressors, which prevent enhancer activity in appropriate places, as seen here for Cic and E(spl)bHLH.

The disparate activities of the *argos* enhancers suggests that correct modes of response will also require functional boundaries to enable the enhancers to function independently. As no insulator elements have been reported within the *argos* intron, based on the binding of known factors such as Su(Hw) and CTCF (44), the mechanism that separates the different functions remains to be elucidated. Other examples of independently functioning enhancers that lack clearly defined insulator elements include the *even-skipped* stripe enhancers. In this context, the activators and repressors bound to each enhancer act only over short distances, and the spacer sequences between the enhancers prevent cross-regulation (40). As spacers of a few hundred base pairs were sufficient to enable the *even-skipped* enhancers to function independently, it is possible that a similar mechanism enables the *argos* enhancers to operate properly. Such independent operation of these context-dependent enhancers is pivotal for their alternate modes of Notch regulation, and it is likely that similar mechanisms operate when genes are required to adopt different response modes to other widely active signaling pathways.
